# The emerging role of zinc transporters in cellular homeostasis and cancer

**DOI:** 10.1038/sigtrans.2017.29

**Published:** 2017-07-28

**Authors:** Elizabeth Bafaro, Yuting Liu, Yan Xu, Robert E Dempski

**Affiliations:** 1 Department of Chemistry and Biochemistry, Worcester Polytechnic Institute, Worcester, Massachusetts, USA; 2 Department of Obstetrics and Gynecology, Indiana University School of Medicine, Indianapolis, Indiana, USA

## Abstract

Zinc is an essential micronutrient that plays a role in the structural or enzymatic functions of many cellular proteins. Cellular zinc homeostasis involves the opposing action of two families of metal transporters: the ZnT (SLC30) family that functions to reduce cytoplasmic zinc concentrations and the ZIP (SLC39) family that functions to increase cytoplasmic zinc concentrations. Fluctuations in intracellular zinc levels mediated by these transporter families affect signaling pathways involved in normal cell development, growth, differentiation and death. Consequently, changes in zinc transporter localization and function resulting in zinc dyshomeostasis have pathophysiological effects. Zinc dyshomeostasis has been implicated in the progression of cancer. Here we review recent progress toward understanding the structural basis for zinc transport by ZnT and ZIP family proteins, as well as highlight the roles of zinc as a signaling molecule in physiological conditions and in various cancers. As zinc is emerging as an important signaling molecule in the development and progression of cancer, the ZnT and ZIP transporters that regulate cellular zinc homeostasis are promising candidates for targeted cancer therapy.

## Introduction

Zinc is the second most abundant transition metal in the human body. Within a cell, zinc is redox-inert and has a valence state of +2. Zinc is maintained in the Zn^2+^ state under all biologically relevant redox potentials and pH conditions.^1^ Zinc shows flexible and dynamic coordination geometry with nitrogen, oxygen and sulfur ligands present in histidine, glutamate, aspartate and cysteine residues in proteins.^1^ Thus, the chemical properties of zinc are ideal for its many biological roles.

The essentiality of zinc as a trace nutrient was first discovered in the fungus *Aspergillus niger* in 1869, but it was nearly a century later that zinc was found to be an essential micronutrient for humans.^2,3^ Zinc deficiency is a global public health problem, leaving ~2 billion people at risk for deficiency of this trace metal.^4^ Human zinc deficiency causes a myriad of symptoms, including diarrhea, alopecia, immune system impairment, chronic inflammation, compromised physical growth and development, neurological deficits and impaired reproductive development.^5,6,7^ In addition to its important role in normal human physiology, zinc has been associated with pathophysiological conditions, including cardiovascular disease, depression, neurodegenerative diseases such as Alzheimer’s disease, diabetes mellitus and cancer.^8^ Consistent with its multifaceted roles in human health and disease, zinc has a number of biological functions at the cellular level. Since it was first discovered as a component of the erythrocyte enzyme carbonic anhydrase in 1939, zinc has been identified as a cofactor in over 300 enzymes spanning all enzyme classes.^9,10^ Equally, zinc is found as a structural component of proteins, and it has been estimated that ~3000 proteins, or 10% of the human proteome, are zinc containing.^11^ Zinc also serves as a signaling molecule that mediates a number of cellular signal transduction processes.^12^

In this review, we will provide an overview of zinc homeostasis in humans, emphasizing the role of zinc transporters and recent developments in the elucidation of their structures. The role of zinc as a signaling molecule will be discussed. In addition, we will examine the interplay of zinc transporters and zinc signaling pathways in several cancer types.

## Zinc homeostasis

The human body contains about 2 g of zinc, of which only 0.1% is present in plasma and the remainder is present within the cells.^13^ In mammalian cells, zinc is in one of two forms: protein-bound zinc, which includes zinc both tightly and loosely bound to proteins; and free zinc ions, which are likely not free but are bound by unknown (non-protein) ligands.^14,15,16^ The free zinc concentration in mammalian cells, while in the picomolar range, likely represents a physiologically significant source of zinc, particularly regarding its role in signaling.^16^ The diverse forms of zinc in the cell make it difficult to assess accurately the intracellular distribution of zinc. Early macro-analytical studies with fractionated rat liver cells, as well as more recent measurements using inductively coupled plasma mass spectrometry on fractionated human lung adenocarcinoma cells, reported that 30–40% of total intracellular zinc is found in the nucleus, 50% of zinc is in the cytoplasm and the remaining 10–20% is membrane-associated.^17^ Nuclear zinc, which is tightly bound to proteins, constitutes the permanently bound zinc pool and is unlikely to be detectable by fluorescent indicators, which measure chelatable, or loosely bound, zinc.^18,19^ Using a fluorescent indicator and organelle-specific dyes, Lu *et al.*^19^ demonstrated that free or transiently bound zinc is found in the endoplasmic reticulum (ER), Golgi apparatus and mitochondria. Newer micro-analytical techniques employing synchrotron X-ray fluorescence chemical imaging allow detection of both loosely and tightly bound metals with high spatial resolution and have demonstrated zinc pools in the nucleus, mitochondria and vesicles.^20,21^ This intracellular compartmentalization of zinc is one of the mechanisms by which cells maintain zinc homeostasis and zinc regulates cellular functions. Cellular zinc homeostasis, both total intracellular zinc and compartmentalized zinc, is tightly controlled by the concerted action of proteins that transport, sense, store and release zinc.

### Zinc transport

Zinc transporters responsible for maintaining cytosolic zinc concentrations, as well as zinc levels in cellular compartments, belong to two solute carrier families, SLC30 and SLC39. The human SLC30, or ZnT, family comprises 10 members (Table 1) that function to decrease the cytosolic concentration of zinc by transporting zinc from the cytosol to the extracellular space or into intracellular compartments. ZnT1 is the only SLC30 member that is ubiquitously expressed on the plasma membrane and, consequently, plays an important role in regulating cellular zinc homeostasis.^22^ Consistent with its essential role, homozygous *Znt1* knockout mice are embryonically lethal.^43^ The human SLC39, or ZIP (Zrt-, Irt-like proteins), family comprises 14 members (Table 2) that function to increase the cytosolic concentration of zinc by transporting zinc into the cytosol from the extracellular space or from intracellular compartments. Currently, there is no direct structural information available for any full-length, human ZnT or ZIP transporter. However, the X-ray crystal and cryoelectron microscopy structures of a bacterial ZnT homolog, YiiP, as well as a structural model of the human ZIP4 and structural information on the extracellular and intracellular domains of ZIP4 provide valuable mechanistic insight into zinc transport by these two families.^77,78,79,80,81,82^

**Figure 1:**
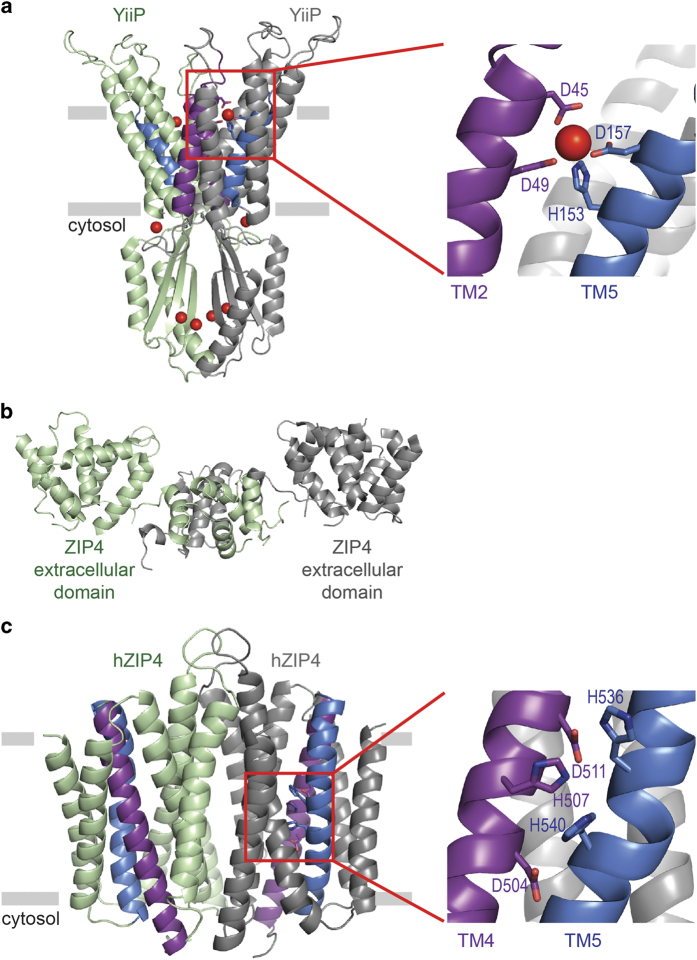
Structural basis for zinc transport by ZnT and ZIP proteins. (**a**) Crystal structure of bacterial ZnT homolog showing zinc (red spheres) bound in the intracellular domain, the membrane interface and TM domain. TM2 (purple) and TM5 (blue) are involved in zinc ion coordination.^78^ (**b**) Crystal structure of mammalian ZIP4 N-terminal ectodomain.^81^ (**c**) Structural model of human ZIP4 TM domain.^80^ TMs 2, 4, 5 and 7 are located in the center of each monomer, and TM4 (purple) and TM5 (blue) contain conserved histidine and aspartic acid residues predicted to coordinate zinc.

X-ray crystal and cryoelectron microscopy structures of the bacterial ZnT homolog YiiP, a proton:zinc antiporter, reveal an alternating access mechanism for zinc transport. YiiP functions as a physiological homodimer, and each protomer consists of six transmembrane (TM) helices, with TMs 1, 2, 4 and 5 forming a helical bundle (Figure 1).^77,78,79^ Within the helical bundle, a zinc ion is tetrahedrally coordinated by three highly conserved aspartic acid residues and one highly conserved histidine in TM2 and TM5.^77^ Mutagenesis experiments demonstrated the important role of this conserved DD–HD zinc-binding site for transport function and metal selectivity.^77,83,84^ The YiiP structures differ in the orientation of the helical bundle with the cryo-EM structure representing the inward-facing conformation and the X-ray crystal structure representing the outward-facing conformation of the transporter.^77,78,79^ Conformational switching is due to reorientation of TM5 that is triggered by zinc binding.^85^ The bacterial YiiP structures provide a model for zinc transport by mammalian ZnT proteins. Mammalian ZnTs are predicted to function as metal:proton exchangers, are predicted to have six TM helices and have a signature metal-binding site in TM2 and TM5, similar to the DD–HD site in the bacterial proteins.^86,87^ The HD–HD motif is responsible for the zinc specificity of ZnTs, as the bacterial DD–HD site allows for zinc and cadmium transport and the ND–HD site in ZnT10 confers manganese specificity.^84,88^

In addition to the TM domain with its zinc coordination site, the bacterial YiiP structures have two large C-terminal intracellular domains (one from each protomer), which, along with two intracellular loops, establish the primary dimerization contact.^77,78^ Human ZnTs are similarly predicted to have large C-terminal cytoplasmic domains, and the C-terminal domain is involved in dimerization for human ZnTs.^89^ ZnT3 and ZnT4 form homodimers via covalent bonds between two tyrosine residues (Y357 and Y372 in ZnT3) in their C-terminal domains, and a YX(XX)(E/D) motif is likely required for dimerization.^89^ Human ZnT1–5, all of which possess the YX(XX)(E/D) motif, form homodimers; whereas ZnT6, which has several C-terminal tyrosine residues but lacks the YX(XX)(E/D) motif, only forms a heterodimer with ZnT5.^89,90,91,92^ ZnT1–4 are also capable of forming heterodimers.^91^ ZnT dimerization is likely required for proper targeting of the transporter to its cellular location and for zinc transport activity.^89^ Molecular modeling of human ZnT3 based on the bacterial structures revealed that dimerization via dityrosine bond formation generates zinc-binding sites that create a putative zinc translocation pathway through the protein.^89^ In the bacterial YiiP structure, a binuclear zinc-binding site was observed in the C-terminal domain and has coordinating ligands contributed by both protomers.^77,78^ The similarity between the YiiP intracellular domain and the βαββαβ metallochaperone-like fold suggests that the cytoplasmic domain of ZnTs may be involved in protein-mediated metal transfer.^77,93^ A third zinc-binding site was identified in the YiiP intracellular loop between TM4 and TM5 and may be involved in transfer of the zinc ion to the membrane zinc-binding site or may function in zinc sensing.^77,78^

Compared to ZnTs, less structural and biochemical information is known about the ZIP family of metal transporters. ZIP family members show a higher diversity, compared to the ZnT family, with respect to metal specificity, transport of zinc, iron, manganese, copper and cadmium.^80,94,95,96^ The energy source for metal translocation by ZIP proteins, while not driven by ATP hydrolysis, remains elusive, and several driving forces, including bicarbonate, pH dependence or phosphorylation, have been proposed.^48,95,96,97,98^ Further, a bacterial ZIP protein reconstituted in proteoliposomes was shown to mediate non-saturable and electrogenic zinc flux, suggestive of zinc channel activity.^99^ This observation led Lin *et al.*^99^ to speculate that ZIP proteins may function as channels, rather than transporters, and the downhill concentration gradient driving zinc influx into the cytosol through ZIP channels is sustained by cellular homeostasis.

The 14 human ZIP family members are classified into four subfamilies (Table 2): ZIPI (with members ZIP1, ZIP2 and ZIP3), ZIPII (with member ZIP9), gufA (with member ZIP11) and LIV-1 (with members ZIP4–8, ZIP10 and ZIP12–14).^50^ All ZIP members are predicted to have eight TM segments, and members of the LIV-I family, with the exception of ZIP13, are predicted to have a large, extracytosolic N-terminal domain. Recently, the N-terminal domain of a ZIP4 protein was solved using X-ray crystallography. Human ZIP4 is expressed in the gastrointestinal tract on the apical surface of enterocytes and as such is the major zinc transporter responsible for the uptake of dietary zinc. Mutations in ZIP4 cause the inherited zinc deficiency disease *acrodermatitis enteropathica* (AE).^55,100^ AE mutations affect the expression, trafficking, uptake activity or posttranslational processing of ZIP4, and a number of these mutations map to the N-terminal extracellular domain.^101,102,103^ The N-terminal domain is posttranslationally cleaved during extended periods of zinc starvation.^102^ The crystal structure of the black fruit bat ZIP4 extracellular domain revealed a homodimer with two subdomains (Figure 1).^81^ The dimer core, comprising the C-terminal subdomain, forms two helix-turn-helix folds and contains the PAL sequence that is highly conserved among the LIV-1 subfamily.^81^ The second subdomain, comprising the N-terminal residues, has a unique α-helical fold.^81^ Both subdomains were shown to be required for optimal zinc transport activity. The subdomains are connected by a disordered linker region that is important for proper folding and trafficking of the full-length ZIP4 protein, as well as for zinc transport.^81^ Analysis of the N-terminal ectodomains of the other LIV-1 family members revealed that the dimerization subdomain is conserved, with the exception of ZIP7 and ZIP13.^81^ The ZIP7 and ZIP13 ectodomain sequences are the most divergent among SLC39 members, and the oligomerization status of the ZIP7 and ZIP13 ectodomains is unknown. As the full-length ZIP7 and ZIP13 proteins form homodimers in the membrane, oligomerization of SLC39 proteins is not mediated exclusively by their ectodomains.^73,98^ The ZIP5 ectodomain has also been purified and shown to be homodimeric.^104^ Interestingly, ZIP5, as well as ZIP6 and ZIP10, colocalize with the prion protein.^104,105^ An evolutionary link between ZIP ectodomains and the prion protein has been suggested but the functional and structural significance remains to be elucidated.^106^ It has been hypothesized that the extracellular domains of ZIP proteins may act as extracellular zinc sensors.^81^

Although to date, there is no crystallographic or cryo-EM structural data that encompasses the membrane domain for any ZIP protein, a computational model of the human ZIP4 protein provides clues as to how ZIP proteins function. Using co-evolution contact predictions and Rosetta *ab initio* structure prediction, structural models of the TM domain of ZIP4 were generated (Figure 1).^80^ The models exhibit eight TM helices with TMs 2, 4, 5 and 7 forming a helical bundle that contains a putative metal permeation pathway.^80^ The putative ZIP4 metal permeation pathway is lined with highly conserved histidine and aspartic acid residues, analogous to the transmembrane zinc-binding site (DD–HD) observed in the crystal structure of the ZnT homolog YiiP (Figure 1).^77,80^ In support of the model’s proposed zinc translocation pathway, the transport kinetics and metal specificity of ZIP4 are altered upon mutagenesis of the conserved histidine residues H507 in TM4 and H536, the first histidine in the conserved HEXXH motif in TM5.^80^ Equally, ZIP8 and ZIP14 have a variation of the HEXXH motif (EEXXH), wherein the glutamic acid substitution is believed to contribute to the broader metal specificity of these SLC39 members.^107^ In generating the ZIP4 structural models, the contact predictions could only be satisfied when ZIP4 was modeled as a dimer with TM helices forming the dimer interface.^80^ Evidence for homodimerization of other ZIP proteins, including ZIP2, ZIP7 and ZIP13, as well as a heteromer of ZIP6 and ZIP10 supports the ZIP4 dimer model.^73,98,108,109^

The ZIP4 structural modeling did not include the large cytoplasmic loop between TMs 3 and 4, which is predicted to be present in all ZIP family members. Studies with the ZIP4 protein have shown that its plasma membrane surface expression is regulated by the cytoplasmic loop in a zinc-dependent fashion. As cytosolic zinc concentrations increase (low micromolar), ZIP4 undergoes endocytosis.^110^ At higher cytosolic zinc concentrations (~20 μM), ZIP4 is ubiquitinated at a conserved lysine residue located on the TM3–TM4 loop and is subjected to proteasomal degradation.^111^ Further studies using the purified protein region have confirmed that the loop directly binds two zinc ions sequentially.^82^ The first zinc is tightly coordinated by a CysHis_3_ site, and the second zinc ion binds with lower affinity to a histidine-only site.^82^ The TM3–TM4 loop is an intrinsically disordered region (IDR) that remains disordered upon zinc binding.^82^ The structural flexibility of intrinsically disordered proteins and IDRs provides a functional advantage by permitting multiple posttranslational modifications, such as phosphorylation and ubiquitination, and interactions with multiple protein partners or ligands.^112,113^ Disordered protein regions tend not to be under strong evolutionary conservation.^114^ Unsurprisingly, the length and amino-acid sequence of the TM3–TM4 loop is not conserved among ZIP proteins, but all contain a variable number of potential zinc-coordinating residues histidine, cysteine, aspartate and glutamate. Accordingly, the large cytoplasmic loop of ZIP transporters likely functions as a protein-specific regulatory region that senses and responds to cytosolic zinc concentrations. The specific protein(s) or ligand(s) interacting with this region have yet to be identified and are likely to be important for our further understanding of ZIP structure–function relationship.

### Intracellular zinc sensing

The metal-response element-binding transcription factor (MTF-1) regulates expression of its target genes in a zinc-dependent manner and thus acts as a cellular zinc sensor.^115,116^ MTF-1 interacts with metal-response element (MRE) sequences in the promoters of zinc-regulated genes.^116^ MTF-1 contains six Cys_2_-His_2_ zinc fingers and three transcriptional activation domains.^116^ In response to zinc, MTF-1 is translocated to the nucleus, where it interacts with MREs and activates transcription of its target genes.^115,116^ Transcription of genes encoding metallothioneins and the zinc efflux transporters ZnT1 and ZnT2 are activated by MTF-1 in response to zinc.^24,115,116^ Recently, the zinc-responsiveness of the human transcriptome was examined when MTF-1 was knocked down relative to normal levels.^117^ Surprisingly, almost 200 genes are differentially expressed in response to zinc when MTF-1 is silenced.^117^ This observation suggests the presence of multiple levels of zinc sensing within the cell, with MTF-1 acting as the master zinc sensor that controls intracellular zinc concentrations via direct effects on gene expression of zinc transporters and metallothioneins.^117^

### Zinc storage and release

Metallothioneins are small, cysteine-rich, metal-binding proteins that maintain metal homeostasis by acting as metallochaperones. Metallothioneins have been shown to act as metal donors and acceptors for metal-containing enzymes and transcription factors.^118,119^ Humans possess four metallothionein isoforms (MT1, MT2, MT3 and MT4) that can maximally bind seven zinc ions via tetrahedral coordination to cysteine residues. The seven zinc ions have been shown, based on structural data, to cluster into two domains, the N-terminal β-domain containing three metal ions and the C-terminal α-domain containing four metal ions. Each zinc-binding site possesses an independent zinc-binding affinity.^120^ Thus, metallothioneins are capable of buffering zinc under physiological conditions and controlling the storage and release of intracellular zinc.

## Zinc signaling

In addition to its structural and functional roles in proteins, zinc acts as a signaling molecule within cells. Early evidence showing zinc-dependent regulatory effects on protein tyrosine phosphatases and transcription factors pointed to the role of zinc in intracellular signaling pathways.^121,122^ However, the work of Yamasaki *et al.*^123^ a decade ago established zinc as a second messenger, whereby changes in its intracellular concentration are directly altered by an external stimulus and relayed to an intracellular signaling axis. Using mast cells, Yamasaki *et al.*^123^ demonstrated that, upon extracellular stimulation of the IgE receptor, zinc is rapidly released into the cytosol from an intracellular store likely originating from the perinuclear and ER regions. The released zinc, termed the ‘zinc wave’, activates two mitogen-activated protein kinase (MAPK) pathways, the extracellular signal-related kinase (ERK) and the c-Jun N-terminal kinase (JNK). MAPKs, such as ERK and JNK, are serine/threonine protein kinases that, upon activation by extracellular or intracellular signals, phosphorylate proteins involved in regulating cell proliferation, differentiation and apoptosis.^124^ In addition to activating MAPK signaling cascades, the intracellular zinc released in the mast cell cytosol inhibits protein tyrosine phosphatase activity.^123^ Protein tyrosine phosphatases, which dephosphorylate tyrosine residues, are involved in many cell signaling cascades, including the integrin and cadherin signaling pathways that control cell adhesion and motility.^125^ The inhibition of protein tyrosine phosphatases by zinc ions occurs at picomolar concentrations of zinc, consistent with cytoplasmic fluctuations of free zinc levels.^126^ Tyrosine phosphorylation via protein tyrosine kinases is also a key component of the cytoplasmic accumulation of zinc that occurred in leukocytes treated with the extracellular stimulus lipopoylsaccharide.^127^ Lipopolysaccharide produces a rapid increase in cytosolic zinc in mammalian leukocytes, and the zinc wave influences signaling pathways involving MAPKs and the transcription factor NF-κB, which targets genes involved in cellular development, growth and apoptosis.^18^

The zinc wave induced by extracellular stimuli in both mast cells and leukocytes occurred rapidly (within minutes) and originated from intracellular zinc stores.^123,127^ This phenomenon has been termed ‘early’ zinc signaling.^128^ In addition to its role as a second messenger in ‘early’ zinc signaling events, zinc has been shown to participate in ‘late’ signaling events that occur hours after stimulation and involve zinc transporters.^128^ One such ‘late’ signaling event was observed during the embryonic development of zebrafish, during which signal transducer and activator of transcription (STAT) proteins are active.^129^ STAT proteins are activated via a signal transduction pathway that is stimulated by extracellular binding of cytokines or growth factors to a receptor. Once activated, STAT proteins transcriptionally regulate a number of genes. In the zebrafish, STAT3 was shown to stimulate transcription of the zinc importer ZIP6. The STAT3-dependent ZIP6 expression results in downstream activation of the transcriptional repressor Snail, which is involved in the epithelial to mesenchymal transition (EMT) in embryonic development.^129,130^ Although the function of ZIP6 in zinc uptake during zebrafish embryonic development was not demonstrated, this study provided the first evidence showing a metal homeostatic mechanism (ZIP6 transporter) is regulated by signal transduction (STAT signaling), and the altered cellular metal homeostasis signals changes in cellular processes. Expression of the zinc exporter ZnT2 has also been shown to be regulated by a STAT signaling pathway, and ZnT2 transcription is stimulated by STAT5.^24,131^

Another zinc transporter, ZIP14, was shown to be involved in a G-protein-coupled receptor (GPCR) cAMP signaling pathway using ZIP14 knockout mice, which displayed growth defects.^132^ The growth defects observed in the ZIP14-deletion mice resulted from inactivation of the parathyroid hormone-related peptide (PTHrP) signaling pathway that involves cAMP. PTHrP activates adenylyl cyclase, which increases cAMP levels. cAMP then stimulates protein kinase A (PKA), resulting in PKA translocation to the nucleus and activation of the cAMP response element-binding (CREB) transcription factor by phosphorylation. Phosphorylated CREB (p-CREB) promotes transcription of *c-fos*, another transcription factor whose target genes are involved in cell differentiation, proliferation, survival and EMT. This signaling pathway is disrupted in the ZIP14 knockout mice, which show decreased nuclear translocation of PKA, a downregulation of p-CREB and lower cAMP levels.^132^ The decreased levels of cAMP are due, not to a decrease in the hormone PTHrP, but to increased activity of phosphodiesterase (PDE). PDEs are known regulators of GPCR signaling pathways. Significantly lower intracellular zinc levels were measured in growth plates from the ZIP14 knockout mice than in ZIP14-expressing mice, demonstrating that ZIP14 is required for zinc uptake and establishing a link between zinc and GPCR/cAMP-dependent signaling cascades.^132^

Expanding the list of signaling cascades influenced by zinc, cellular zinc status directly affects the activity of the STAT pathway.^133^ STAT proteins regulate the expression of genes involved in cell proliferation, differentiation, survival and apoptosis.^134^ The STAT signaling pathway begins with an extracellular signal that activates the Janus tyrosine kinase, which then activates STAT proteins by phosphorylation. Phosphorylated STAT proteins are translocated to the nucleus where downstream signals are regulated transcriptionally. Levels of phosphorylated STAT1 and STAT3 are 40–50% lower, and corresponding nuclear levels of these STAT proteins are lower, in fetal rat brains upon maternal zinc deficiency compared to the zinc-adequate controls.^133^ Equally, cytosolic zinc accumulation in lactating ZnT2 knockout mice resulted in a significant reduction in phosphorylated STAT5 levels, but not total STAT5 levels, compared to mice expressing ZnT2.^135^ As zinc has been shown to influence many phosphorylation-dependent signaling cascades (for example, MAPKs, protein tyrosine phosphatases and transcription factors) that play important roles in cell development, proliferation and cell death, proper maintenance of cellular zinc homeostasis is critical. The dysregulation of cellular zinc homeostasis and its detrimental effects on intracellular signaling have many pathophysiological consequences.

Compared to the best-studied metal second messenger calcium, a more comprehensive picture of zinc cellular signaling remains elusive. Technological advancement in zinc measurement and regulation, as well as more systematic signaling studies are critical in making progress in these areas. In the following section, the consequences of zinc dyshomeostasis on zinc signaling in cancer will be discussed.

## Zinc transporters and zinc signaling in cancer

Cellular zinc homeostasis is altered in cancer. Zinc levels in serum are typically decreased in patients with tumors, including breast, head and neck, lung, liver, prostate, pancreatic and gynecological tumors, compared to normal serum levels.^136^ However, while serum zinc levels are low in most cancers, studies measuring zinc levels in tumor and/or tumor peripheral tissues in different solid cancer types are inconsistent, reporting elevated, decreased or no change in zinc levels when compared with the corresponding normal tissues.^136^ Although the mechanism and effects of zinc dyshomeostasis on cancer initiation and progression are not fully defined, some studies have reported changes in zinc transporter status and effects of altered zinc levels on signaling pathways in tumor tissues and cancer cell lines. The most extensive studies on zinc homeostasis and zinc signaling in cancer have focused on pancreatic, prostate and breast tumors.

### Pancreatic cancer

The pancreas, a gland adjacent to the stomach, has two distinct functionalities as an endocrine and an exocrine gland, and proper pancreatic function requires physiological zinc homeostatic mechanisms. As an endocrine gland, the pancreatic islet cells, which include α, β and δ cells, contribute to blood glucose levels and insulin production. The α cells secrete the hormone glucagon to control hypoglycemia, and zinc acts as a signaling molecule for glucagon secretion. The zinc importers ZIP1, ZIP10 and ZIP14 are expressed on the plasma membrane of pancreatic α cells.^29^ Pancreatic β cells produce a zinc:insulin complex that is stored in zinc-rich secretory granules.^39^ ZIP4 is expressed on the plasma membrane of pancreatic β cells, suggesting that ZIP4 contributes to maintaining adequate zinc levels in the β cells for insulin secretion.^29^ ZIP6 and ZIP7 have also been shown to be expressed in human β cells.^58^ Decreasing the expression level of ZIP6 and ZIP7 changed the uptake of zinc into β cells and decreased insulin secretion. In addition, the zinc exporter ZnT8 transports zinc into the β-cell granules, providing zinc for the zinc:insulin complex.^39^ As an exocrine gland, pancreatic acinar cells generate digestive enzymes, many of which are zinc-dependent, in zymogen granules. The SLC30 member ZnT2 transports zinc from the cytosol into the zymogen granules.^24^ The zinc importer ZIP5 is expressed on the plasma membrane of the acinar cells.^137^

Pancreatic cancer is ranked as the fourth leading cause of death due to cancer, and one- and five-year survival rates are 20 and 7%, respectively. Dysregulation of cellular zinc homeostasis has been implicated in the progression of pancreatic cancer. Early studies identified increased mRNA levels of the zinc transporter ZIP4 in 16 of 17 human pancreatic adenocarcinoma samples compared to normal pancreatic tissue.^138^ Equally, in mouse models, ZIP4-expressing xenografts yield larger tumors and increased cell proliferation compared to negative controls.^138^ ZIP4 localizes to the basolateral membrane of pancreatic β cells, and its upregulation in pancreatic tumors would presumably increase cellular zinc levels.^138,139^ However, using zinc-sensitive histochemical staining, Costello *et al.*^140^ measured lower zinc levels in human pancreatic cancer tissues spanning early to late malignant stages compared to normal tissues. Concurrent with the decreased zinc levels, ZIP3 is downregulated in the pancreatic cancer tissues, but ZIP4 levels were not quantified.^140^ In a comparative study of the mRNA levels of all ZIP and ZnT transporters in human pancreatic cancer and normal tissues, all ZIP proteins are downregulated in the cancer tissues with the exception of ZIP4, which is upregulated.^141^ Similarly, gene expression levels for all ZnT transporters are lower or unchanged in the pancreatic cancer tumors compared to normal tissues.^141^ Although the data are consistent with the previously observed downregulation of ZIP3 and upregulation of ZIP4, it is difficult to reconcile how increases in zinc transporter mRNA levels result in the decreased cellular zinc levels observed in pancreatic cancer tissues. It should be noted that, to date, there has been no comprehensive study comparing cellular zinc concentrations and zinc transporter expression in the same tissue samples. High variability in zinc transporter expression has been observed in different pancreatic cancer tissues and cell lines.^141^ Equally, mRNA levels and protein expression do not necessarily correlate as many zinc transporters, including ZIP4, are regulated by posttranslational mechanisms.^142^ Moreover, as ZIP4 has been shown to transport other metals, including iron and copper, it remains to be determined whether the observed increase in ZIP4 expression in pancreatic cancer alters cellular zinc concentrations or alters the cellular homeostasis of another metal.^80,94^ For example, serum copper levels are higher in patients with pancreatic cancer, and copper has been shown to have tumor-promoting activity in cells.^143,144,145, 146^

### Prostate cancer

The human prostate gland contains exceptionally high levels of zinc (~800–1500 μM in prostate epithelial cells compared to ~100–500 μM in other soft tissue cells), and the zinc concentration in prostatic fluid is about 500-fold greater than in plasma.^147^ Also, the mitochondrial zinc level in prostate cells is higher than in other cell types.^147^ Zinc accumulation in prostate epithelial cells is responsible for their unique metabolic and energetic status as ‘citrate producing’, ‘low respiring’ cells.^147^ Zinc disrupts both the Kreb’s cycle, by inhibiting *m*-aconitase, which converts citrate to *cis*-aconitate resulting in citrate accumulation, and mitochondrial electron transport, likely via inhibition of cytochrome *c* reductase. Zinc accumulation in prostate epithelial cells is attributed to the expression of ZIP1, which is present on the basolateral membrane and functions as the major importer of zinc from circulating blood plasma into prostate cells.^44,148^ In addition to ZIP1, ZIP2 and ZIP3 have been identified on the apical surface of normal prostate cells, and it is speculated that these proteins function in the reuptake of zinc from the prostatic fluid.^149^

Compared with normal or nonmalignant prostate tissue, malignant prostate tissue displays markedly lower levels of zinc, and the decline in zinc occurs early in the malignant transformation.^150^ Concomitant with the declining zinc levels, ZIP1 gene expression is downregulated in prostate cancer.^151^ Other ZIP family members, ZIP2, ZIP3 and ZIP4, also show reduced expression in prostate cancer cells.^149,152^ In addition, ZIP3 localizes to lysosomal membranes in a prostate cancer cell line compared to its plasma membrane localization in normal cells.^148^ Changes in the expression levels of the zinc efflux transporters ZnTs during prostate tumorigenesis are ambiguous. ZnT1 expression has been reported to be decreased, increased or unchanged, ZnT4 is decreased or unchanged, ZnT10 is increased and ZnT5 and ZnT6 are decreased in prostate cancer tissues and cell lines compared to normal or nonmalignant prostate tissue.^153,154,155,156,157^ Although it is unclear what role ZnTs play in prostate cancer, the important role of ZIP1 in normal prostate epithelial cells and in prostate cancer development led to the designation of ZIP1 as a tumor suppressor gene in the prostate gland.^150^ As prostate cancer cell lines constitutively express ZIP1, as well as other zinc transporters, it is hypothesized that the downregulation of zinc transporters in malignant prostate tissue is due to epigenetic silencing.^150^

**Figure 2:**
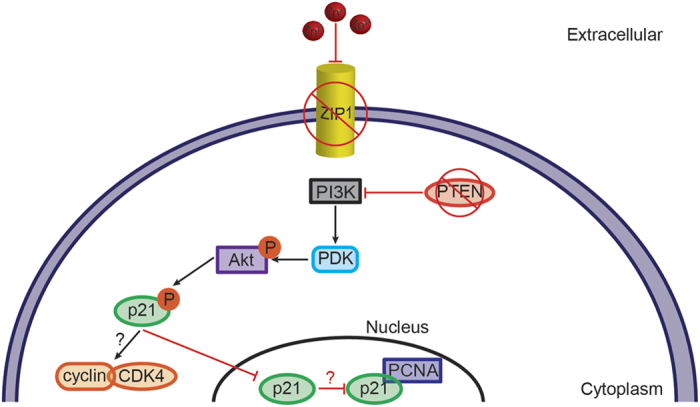
Zinc deficiency in prostate cancer cells promotes cell survival via a phosphoinositide 3-kinase (PI3K) signaling pathway. Prostate cancer cells lack ZIP1 and PTEN. In zinc-deficient, PTEN-deficient conditions, PI3K is stimulated, leading to phosphorylation of protein kinase B (Akt) via phosphoinositide-dependent kinase (PDK). p-Akt then phosphorylates the cyclin-dependent kinase inhibitor p21. Phosphorylated p21 is retained in the cytoplasm where it may affect the cell cycle activity of the cyclin D/cyclin-dependent kinase 4 (CDK4) complex. In addition, the lack of nuclear p21 may prevent activation of the PCNA, which normally prevents DNA synthesis during cell cycle progression. Thus, zinc deficiency affects p21 signaling pathways, leading to cell proliferation in prostate cancer cells.

The reduced zinc levels in prostate tumors caused by decreased ZIP transporter expression alter intracellular zinc signaling pathways leading to tumor-promoting activities. Zinc status affects signaling via the Akt protein (protein kinase B) in malignant prostate cells (Figure 2).^158^ Zinc-deficient prostate cancer cells accumulate phosphorylated Akt (p-Akt) due to the absence of the phosphatase and tensin homolog (PTEN) protein. PTEN is known to be inactivated by genomic deletion or rearrangements in the early stages of prostate cancer oncogenesis.^159,160^ Normally, PTEN negatively regulates phosphoinositide 3-kinase (PI3K) activity. In the absence of PTEN, PI3K phosphorylates Akt, and p-Akt subsequently phosphorylates the cyclin-dependent kinase inhibitor p21.^158^ Phosphorylated p21 is retained in the cytoplasm, affecting cell cycle regulation likely due to downstream effects on the cyclin D/cyclin-dependent kinase 4 (CDK4) complex and the proliferating cell nuclear antigen (PCNA). Thus, an Akt-p21 signaling pathway promotes cell proliferation in prostate cancer cells, and this pathway depends on the absence of the zinc transporter ZIP1 and PTEN. PTEN also interacts with MTF-1, linking PTEN activity to the regulation of MTF-1 target genes, such as metallothioneins.^161^ Metallothioneins, particularly MT1 and MT2, are downregulated in prostate cancer tissues compared to normal prostate tissue.^162,163^ Moreover, the levels of metallothionein in human prostate cancer tissue inversely correlate with prostate cancer relapse.^163^ Accordingly, prostate cancer exhibits a complex network linking cellular zinc homeostatic mechanisms, including zinc transporters and metallothioneins, to signaling pathways that promote tumorigenesis.

### Breast cancer

The cellular zinc homeostatic mechanisms in the normal human mammary gland are not known precisely, and much of what is known has been elucidated from human models or mouse studies.^164^ Zinc plays an important role in normal mammary gland growth and development and during lactation and post-lactation transformations (as reviewed by McCormick *et al.*).^164^ Normal mammary epithelial cells express ZIP5 at the cell surface where ZIP5 is likely involved in metal uptake.^164^ Other ZIP proteins, including ZIP3, ZIP7, ZIP11, ZIP12 and ZIP14, are expressed within intracellular compartments.^68^ ZnT2 also plays an important role in mitochondrial zinc transport during normal mammary gland development.^165^ During lactation, the mammary gland hyperaccumulates zinc, particularly in the Golgi apparatus and the secretory system, to meet increased cellular needs and for secretion into breast milk.^166^ ZIP5, ZIP8 and ZIP10 may be involved in zinc uptake from plasma during lactation.^68^ Interestingly, ZIP7, which exports zinc from the Golgi apparatus and ER, is more highly expressed during lactation, suggesting that the zinc accumulated in the Golgi apparatus is remobilized to the cytosol during lactation.^68^ ZnT4 is also located on the Golgi apparatus and has been suggested to contribute to zinc export into breast milk.^31^ Zinc secretion into breast milk requires ZnT2, which localizes both to the endosomal/secretory vesicles and to the plasma membrane.^23^ Mutations in ZnT2 cause the disorder transient neonatal zinc deficiency, confirming its essential role in zinc secretion into breast milk.^33,167^ ZIP3 may function in reuptake of zinc from breast milk.^168^ Finally, post-lactational changes in the mammary gland involve both lysosomal-mediated cell death and mitochondrial-mediated apoptosis.^164^ The zinc requirements for these processes may rely on changes in ZnT2, ZnT4 or ZIP8 localization as these proteins have been shown to localize to lysosomal compartments in other cell types.^31,164,169,170^ Given the importance of zinc homeostasis in the mammary gland, it has been suggested that nutritional zinc status may be a risk factor for breast cancer.^164,171^ In support of this, the incidence of mammary tumors in primiparous mice exposed to a carcinogen is lower in mice fed a zinc adequate diet than in mice fed a marginally zinc-deficient diet, similar to the zinc-deficient diets often consumed by humans.^172^

In contrast to most solid tumors, breast cancer tissues show significantly higher zinc levels compared with normal breast tissue.^136,173,174,175, 176^ Zinc distribution and zinc transporter levels show distinct profiles in breast cancer subtypes.^142^ Breast cancer is classified into basal (or triple negative), human epidermal growth factor receptor 2 (HER2) overexpression and luminal (or estrogen receptor positive, ER+) A (low-grade) and B (high-grade) tumors. Using X-ray fluorescence microscopy, Chandler *et al.*^142^ observed high zinc accumulation around the margins of luminal tumors, whereas basal tumors show uniform distribution of zinc. Breast tumor tissues were analyzed for gene expression of metallothioneins and zinc transporters (ZIP and ZnT). Compared to luminal (ER+) and HER2 overexpression tumors, basal breast cancer tumors expressed higher levels of metallothioneins, *ZIP4* and *ZIP14* genes and lower levels of *ZIP6*, *ZIP9* and *ZIP11*.^142^ Luminal breast cancer tumors express higher levels of the *ZIP6* gene compared to basal and HER2 overexpression tumors, and *ZIP8* gene expression is greater in high-grade versus low-grade ER+ tumors.^142^ Similarly, gene and protein expression profiles for zinc transporters have been assessed in luminal and basal breast cancer cell lines. Kagara *et al.*^66^ observed higher gene expression of ZIP10 in basal cells and higher gene expression of ZIP6 in luminal cells. At the protein level, basal cells display higher levels of ZnT1, ZIP1 and ZIP10 compared to normal cells; luminal cells display higher levels of all ZnT proteins ZIP3, ZIP5, ZIP6, ZIP8 and ZIP10.^142^ It has been suggested that the differing zinc transporter profiles and their effects on cellular zinc homeostasis may contribute to the phenotypic differences, including invasiveness, metastasis and treatment effectiveness, in breast cancer subtypes.^142^

**Figure 3:**
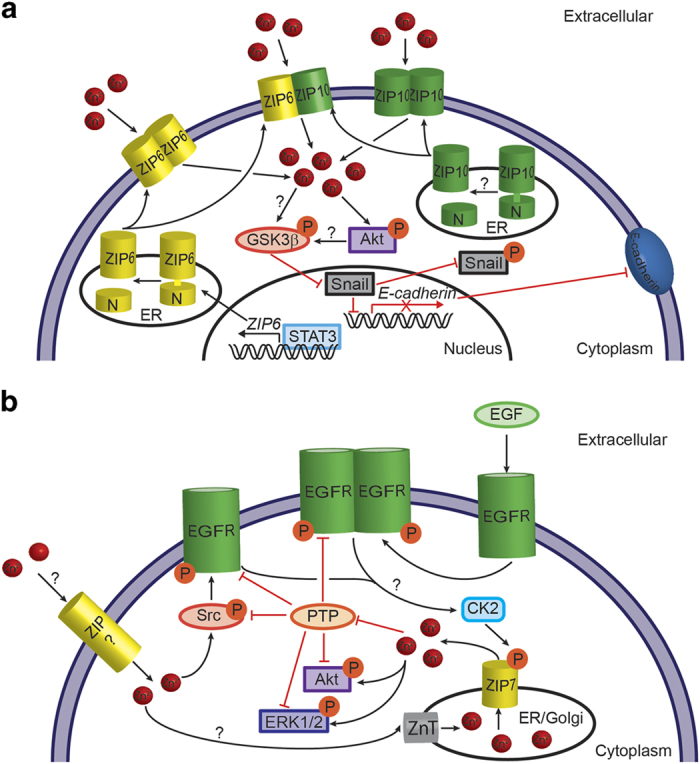
ZIP-mediated signaling pathways promote cell proliferation and metastasis in luminal and tamoxifen-resistant breast cancer cells. (**a**) ZIP6/ZIP10-mediated signaling pathway in luminal breast cancer cells. ZIP6, whose gene expression is induced by STAT3, is expressed on the ER membrane. On cleavage of its N-terminal domain, ZIP6 translocates to the plasma membrane. ZIP10 may undergo the same posttranslational processing leading to plasma membrane localization. On the plasma membrane, ZIP6 and ZIP10 exist as homo- and heteromers. Zinc uptake via ZIP6 and/or ZIP10 results in the inactivation of glycogen synthase kinase-3β (GSK3β) by phosphorylation either directly or indirectly by activation (phosphorylation) of Akt. Inactive GSK3β is unable to phosphorylate the transcription factor Snail, resulting in nuclear retention of Snail. Snail represses transcription of the adhesion molecule E-cadherin, leading to cell migration and metastasis. (**b**) ZIP7-mediated signaling pathway in tamoxifen-resistant breast cancer cells. The signaling pathway involves stimulation of the EGFR, a receptor tyrosine kinase that responds to binding of the extracellular ligand by dimerization, trans-phosphorylation and tyrosine kinase activation. EGFR is also transactivated by the protein tyrosine kinase Src, and this transactivation is stimulated by zinc. It is unclear where the zinc that stimulates Src activates originates, but it may involve a plasma membrane ZIP transporter. EGFR activation results in phosphorylation of ZIP7 by the serine/threonine protein kinase CK2, although it is unclear how the signal is transduced from EGFR to CK2. Phosphorylated ZIP7 transports zinc from the Golgi or ER into the cytoplasm. The increased cytoplasmic zinc inhibits protein tyrosine phosphatases (PTP), causing sustained activation of EGFR and Src. In addition, the released zinc activates kinases ERK1, ERK2 and Akt, which regulate signaling pathways leading to cancer cell proliferation and migration.

ZIP10 and ZIP6 have been linked to tumor migration in both basal and luminal breast cancers.^66,109,177^ In basal breast cancer cells, ZIP10 functions as a zinc importer, and ZIP10-mediated zinc uptake stimulates cell migration.^66^ Similarly, in luminal breast cancer cells, ZIP10 promotes cell migration.^109^ Further, in luminal breast cancer cells, ZIP10 has been shown to form a heteromer with ZIP6.^109^ In both ZIP10- and ZIP6-expressing luminal breast cancer cells, the glycogen synthase kinase signaling pathway is inhibited, leading to activation of the Snail transcription factor that represses genes for cell adhesion and promotes EMT (Figure 3).^109,177^ ZIP6, whose gene expression is induced by STAT3 and in the absence of ZIP10, was observed to undergo cleavage of its N-terminal domain, which alters its cellular localization from the ER to the plasma membrane.^109,177^ As both ZIP6 and ZIP10, along with ZIP5, have a prion-like ectodomain, these data suggest a relationship between this subclass of LIV-1 transporters and breast cancer metastasis.

The most common treatment for luminal (ER+) breast cancers is the anti-estrogen drug tamoxifen. However, almost all patients with metastatic breast cancer and 40% of patients receiving tamoxifen as an adjuvant therapy experience cancer relapse due to acquired tamoxifen resistance (TamR).^178^ An association between TamR and zinc dyshomeostasis has been identified (Figure 3).^179^ Compared to tamoxifen-sensitive cells, TamR breast cancer cells have twice the level of intracellular zinc and show increased mRNA and protein expression levels of the LIV-1 zinc transporter, ZIP7.^179^ The increased intracellular zinc is a direct result of the ZIP7 protein, as a decrease in zinc was observed in TamR cells in which ZIP7 was silenced.^179^ ZIP7, which localizes to the Golgi and ER membranes, is activated by protein kinase CK2 through phosphorylation of serine residues located in the large cytosolic loop of the transporter.^98^ The CK2 stimulation of ZIP7 occurs within minutes after exposure to an extracellular stimulus, suggesting that ZIP7 generates a zinc wave that is transduced to ‘early’ zinc signaling pathways in TamR breast cancer cells.^98^ In TamR cells, zinc stimulates MAPKs (ERK1 and ERK2) and Akt, indicating the involvement of ZIP7-mediated zinc transport in intracellular signaling pathways that affect cell proliferation, cell migration and apoptosis.^98,179^

Similarly, zinc activates the ERK and Akt kinase signaling pathways in breast cancer cells modeling HER2 overexpression tumors.^180^ In the HER2 overexpression cells, ERK and Akt activation arise from a zinc-dependent stimulation of the G protein-coupled estrogen receptor (GPER). Following stimulation, GPER transactivates the receptor tyrosine kinase epidermal growth factor receptor (EGFR). EGFR phosphorylation leads to downstream signaling that stimulates the kinases Erk1, Erk2 and Akt. Phosphorylated Erk1, Erk2 and Akt are translocated to the nucleus where they regulate the transcription of genes involved in cell proliferation, cell cycle progression and migration. The involvement of zinc as a signal for the GPER pathway may have prognostic effects. Higher GPER expression has been linked to poor survival in HER2-positive breast cancer cases.^181,182^

## Conclusion

Recently, zinc has been recognized as a critical signaling molecule in normal cell physiology as well as in pathophysiological conditions, such as cancer. Under normal conditions, zinc has been shown to regulate several phosphorylation-dependent signaling cascades, including MAPKs and Akt, that play important roles in cell development, proliferation and cell death. Many of these same signaling cascades have been shown to be affected by zinc levels in cancer and lead to cancer cell proliferation and metastasis. Zinc signaling is controlled by cellular zinc homeostatic mechanisms that regulate intracellular zinc concentrations, especially the two zinc transporter families ZIP and ZnT. Not surprisingly, altered expression of these transporters is evident in many cancers. As a deeper understanding of the relationship between zinc transporter expression and downstream signaling pathways in tumors is gained, it may become possible to utilize zinc transporter profiles to tailor cancer treatments more effectively.

The complex interplay between zinc transporters and zinc signaling is just beginning to be deciphered. More structural studies on mammalian ZnT and, more importantly, on mammalian ZIP proteins are needed to clarify better the roles of metal transporters in cellular zinc homeostasis. One area that remains unclear is how the zinc signal is relayed from the transporter to the signaling pathways, many of which also involve membrane receptors. As very little free zinc exists in the cell, one area that needs exploration is the identification of protein–protein interactions that may be involved in the zinc relay. A detailed understanding of cellular zinc homeostasis and signaling is not only vital to understanding the role of zinc in normal cellular processes, but may also uncover new targets for cancer drug development.

## Figures and Tables

**Table 1:** Human ZnT proteins

*Protein*	*Major location of protein expression*	*Subcellular localization*
ZnT1	Ubiquitous	Plasma membrane^22^
ZnT2	Mammary gland, pancreas, prostate, retina, intestine, kidney^23,24,25,26,27^	Endosome, lysosome, secretory vesicle, plasma membrane^23,24^
ZnT3	Brain, pancreas, testis^28,29^	Synaptic vesicles^30^
ZnT4	Mammary gland, placenta, prostate, kidney, brain^25,26,31,32,33,34^	Endosome, secretory vesicle, plasma membrane^26,31^
ZnT5	Heart, placenta, prostate, ovary, testis, intestine, thymus, bone^25,32,35^	Golgi, vesicles, plasma membrane^36^
ZnT6	Brain, lung, intestine^34,37^	Golgi, vesicles^37^
ZnT7	Intestine, stomach, pancreas, prostate, placenta, testis, retina, muscle^25,32,38^	Golgi, vesicles^38^
ZnT8	Pancreas, adrenal gland, thyroid, testis^39,40^	Secretory granule^39^
ZnT9	Brain, muscle, kidney^41^	ER^41^
ZnT10	Brain, retina, liver^42^	Golgi, plasma membrane^42^

**Table 2:** Human ZIP proteins

*Protein*	*Sub-family*	*Major location of protein expression*	*Subcellular localization*
ZIP1	ZIPII	Prostate, small intestine, kidney, liver, pancreatic α cells^29,44,45,46^	Plasma membrane, ER^46,47^
ZIP2	ZIPII	Prostate, uterine, epithelial cells, ovary liver, skin^48,49^	Plasma membrane^50,51^
ZIP3	ZIPII	Testes, pancreatic cells^52^	Plasma membrane^53^
ZIP4	LIV-1	Small intestine, stomach, colon, cecum, kidney, pancreatic β cells^29,54^	Plasma membrane^55^
ZIP5	LIV-1	Liver, kidney, spleen colon, pancreas^56^	Plasma membrane^56^
ZIP6	LIV-1	Testis, pancreatic β cells^57,58^	Plasma membrane^57^
ZIP7	LIV-1	Brain, liver, pancreatic β cells^58,59^	Golgi, ER^60^
ZIP8	LIV-1	Pancreas, placenta, lung, liver, testis, thymus, red blood cells^57,61,62^	Plasma membrane, lysosome, endosomes^61^
ZIP9	ZIPI	Prostate^63^	Golgi^64^
ZIP10	LIV-1	Testis, kidney, breast, pancreatic α cells, red blood cells^29,57,62,65,66^	Plasma membrane^57,67^
ZIP11	gufA	Mammary gland, testis, stomach, ileum and cecum^68,69^	Golgi^68^
ZIP12	LIV-1	Neurons, endothelial, smooth muscle and interstitial cells^70,71^	Unknown
ZIP13	LIV-1	Retinal pigment epithelial cell line, osteoblasts^27,72^	ER^73^
ZIP14	LIV-1	Liver, heart, placenta, lung, brain, pancreatic α cells^29,74^	Plasma membrane, mitochondria, endosome, lysosome^75,76^
